# Socioeconomic and environmental patterns behind H1N1 spreading in Sweden

**DOI:** 10.1038/s41598-021-01857-4

**Published:** 2021-11-18

**Authors:** András Bota, Martin Holmberg, Lauren Gardner, Martin Rosvall

**Affiliations:** 1 Integrated Science Lab, Department of Physics, Umeå University, 90187 Umeå, Sweden; 2 Embedded Intelligent Systems Lab, Department of Computer Science, Electrical and Space Engineering, Luleå University of Technology, 97187 Luleå, Sweden; 3 Department of Civil and Systems Engineering, Johns Hopkins University, Baltimore, MD 21218 USA

**Keywords:** Risk factors, Influenza virus, Applied mathematics, Computer science

## Abstract

Identifying the critical factors related to influenza spreading is crucial in predicting and mitigating epidemics. Specifically, uncovering the relationship between epidemic onset and various risk indicators such as socioeconomic, mobility and climate factors can reveal locations and travel patterns that play critical roles in furthering an outbreak. We study the 2009 A(H1N1) influenza outbreaks in Sweden’s municipalities between 2009 and 2015 and use the Generalized Inverse Infection Method (GIIM) to assess the most significant contributing risk factors. GIIM represents an epidemic spreading process on a network: nodes correspond to geographical objects, links indicate travel routes, and transmission probabilities assigned to the links guide the infection process. Our results reinforce existing observations that the influenza outbreaks considered in this study were driven by the country’s largest population centers, while meteorological factors also contributed significantly. Travel and other socioeconomic indicators have a negligible effect. We also demonstrate that by training our model on the 2009 outbreak, we can predict the epidemic onsets in the following five seasons with high accuracy.

## Introduction

A novel pandemic emerged in Mexico during the spring of 2009, caused by a recombined influenza strain derived from circulating swine influenza strains. It spread quickly to other continents and struck Europe with a spring and summer wave, affecting several countries. The rate of transmission subsided as the summer progressed but accelerated again during the autumn season, this time in all European countries. The spread followed a west to east progression, a typical pattern for seasonal influenza^1^.

The first cases in Sweden appeared late in April, and local transmission followed mainly in the major cities. In late September and early October, a nationwide outbreak started, peaking in November. From mid-May, the Swedish Institute for Infectious Disease Control required mandatory reporting of individual cases, which later changed to hospitalized patients supplemented by laboratory notification of positive samples^2^. WHO declared the end of the pandemic on 10 August 2010. However, the mandatory reporting of the H1N1 pandemic strain continued in Sweden during the following five seasons, providing a detailed and thorough collection of epidemic data on a fine spatial resolution.

The spatial component of many infectious diseases is crucial for the understanding of epidemic transmission. Recent years have seen a surge in mathematical modeling to describe the geographic transmission of infectious diseases^3–7^. Specifically, with the increasing availability of geo-tagged epidemiological data, the 2009 A(H1N1) pandemic influenza has been studied using spatially explicit models. For example, studies in the US have revealed how important human mobility patterns are for explaining influenza epidemic transmission^8–11^. A previous geographic study of the pandemic spread over Sweden indicated a progression from the north to the south during 2009^12^. Morris et al.^13^ investigated the relationship between the spatio-temporal spreading of seasonal influenza and demographic, geographic and climatic factors. However, their study focused on Norway and only conducted a joint analysis of the other Nordic countries on the county level.

Meteorological factors associated with the rate of influenza transmission among individuals include precipitation, humidity, temperature, and sun radiation^12,14–16^. However, uncertainties in the data remain, and meteorological drivers may play more significant roles in some geographic regions than others. The role of population size and density in cities is also known to affect the dynamics of influenza transmission and the weight of environmental factors^8,16^. Apart from the importance of schoolchildren for influenza transmission^17^, socioeconomic factors important for influenza spread remain sparsely studied^18^.

In this paper, we seek to uncover the critical socioeconomic, travel, and meteorological factors behind H1N1 outbreaks in Sweden, covering six years between 2009 and 2015, focusing on the start of the epidemic phase. We aim to identify the relationship between the timing of this event and the above factors available for each of the 290 municipalities of Sweden. We also explore the predictive capabilities of our approach by using the 2009 pandemic to train our model and measuring its performance on data from the following years. We analyze Sweden at a much higher resolution compared with previous studies^13^ and extend the potential list of risk factors.

We analyze disease spreading dynamics with the Generalized Inverse Infection Model (GIIM)^19,20,23^, a network-based optimization method. In this model, each municipality is a node, and the edges between nodes represent possible disease spreading paths. The primary output of this model is the set of estimated transmission probabilities between the nodes (geographical areas) of the model. These probabilities can be formalized as a function of known attributes: properties of the nodes (municipalities) and edges (pairs of municipalities) of the network. We estimate the parameters of this function with the Fully Informed Particle Swarm Optimization Method^21^. GIIM can be used with any monotonic infection model, including members of the SEIR family^20,22^. Since our goal is to estimate the timing of the epidemic onset, we select the SI infection model^22^ to represent spreading dynamics. The SI model has been used in literature when modeling the first occurrence of a disease and spreading dynamics during the early phase of an outbreak^23,24^. The epidemic onset falls into the latter category.

We use GIIM to estimate the transmission risk between geographical areas, the importation and exportation risks for all municipalities, and to establish the relationship between the properties of the outbreak and several socioeconomic, travel and meteorological indicators. In addition, we can use the risk factors, their assigned weights, and the attribute function to simulate an outbreak during any given time period, allowing us to predict the timing of the epidemic onset in future seasons. We demonstrate this by training our model on data from the 2009 pandemic and predicting the timing of the onset in later seasons with good accuracy.

## Data

Our analysis covers Sweden on the geographical level of its 290 municipalities. Almost all the data sources are domestic and are available either on the municipality level (only the cities of Stockholm and Göteborg comprise more than one municipality) or the DeSo (demographic statistical areas)^25^ level, a subdivision of the municipality level. The only exception is the weather-station based meteorological data, which we have converted from specific geographic coordinates to the municipality level. Because Sweden covers a large geographical area with significant variance in features, climate, and population, the selected indicators may vary significantly.

### Maps and geographical data

**Figure 1:**
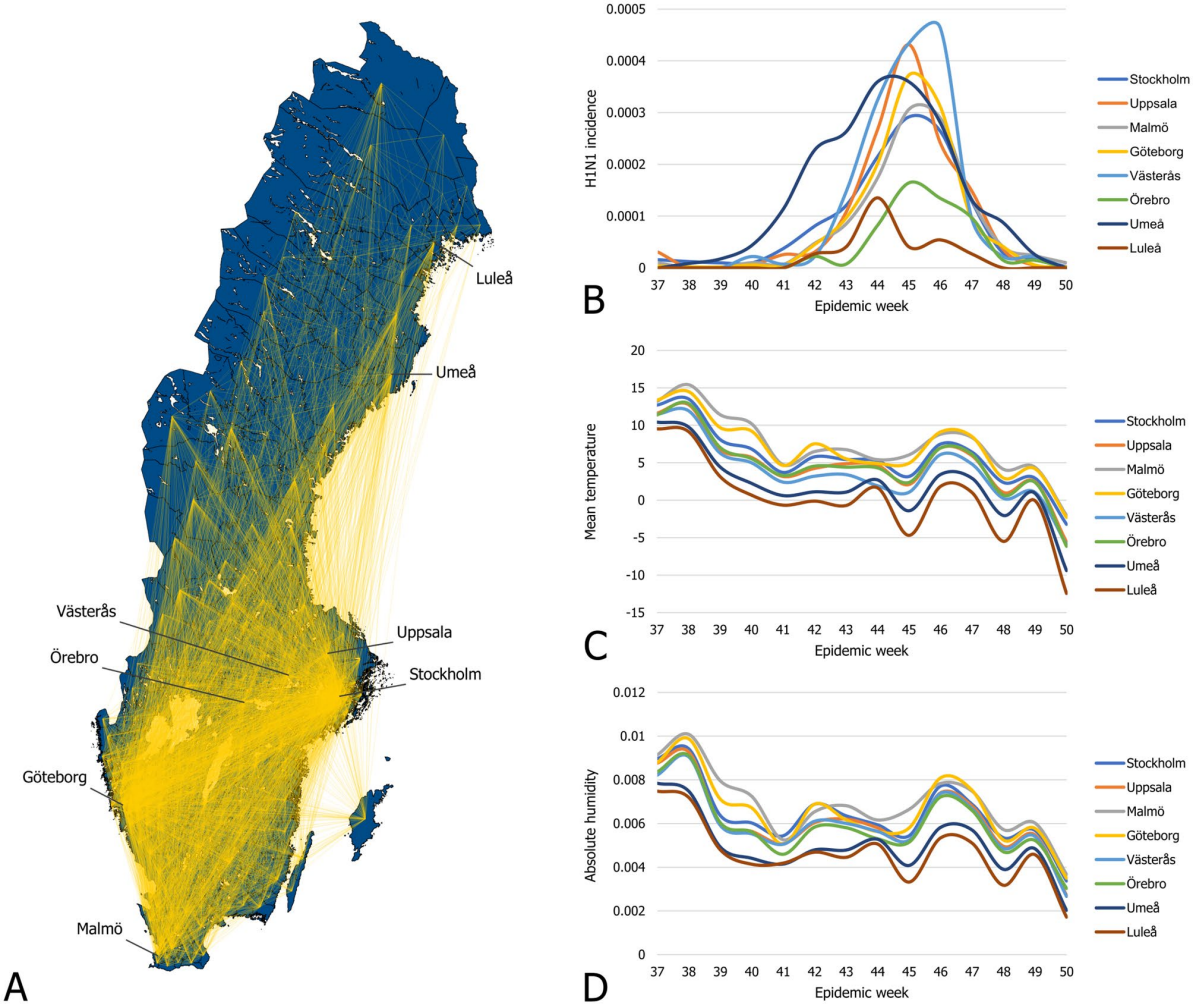
(**A**) The travel network of Sweden. Municipalities on (**B**–**D**) are highlighted. (**B**) Population normalized H1N1 incidence in epidemic weeks 37 to 50 in the municipalities listed on the right side. (**C**) The average mean temperature from week 37 to 50 in the municipalities listed on the right side (shown in celsius). (**D**) The average absolute humidity from week 37 to 50 in the municipalities listed on the right side.

**Figure 2:**
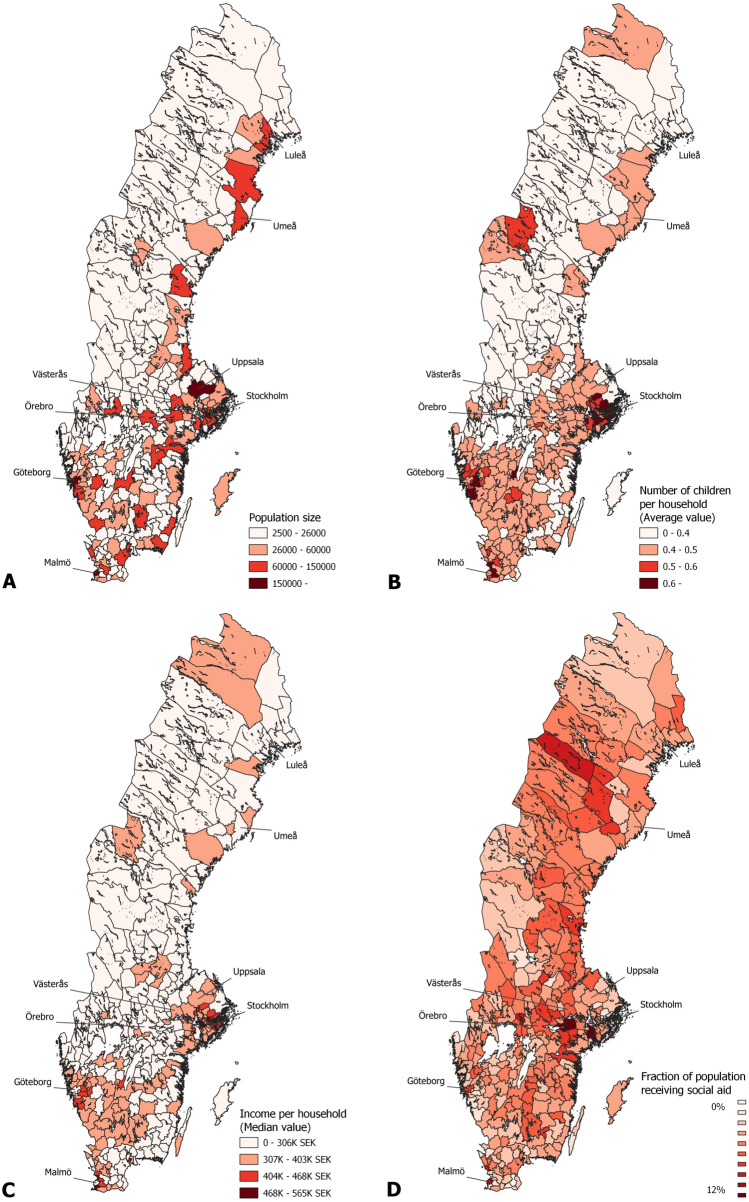
(**A**) Geographical distribution of population size of the municipalities of Sweden. (**B**) Geographical distribution of the average number of children per household in the municipalities of Sweden. (**C**) Geographical distribution of median household income in the municipalities of Sweden. (**D**) Geographical distribution of the fraction of people receiving social aid in the municipalities of Sweden. Municipalities shown on Fig. 1 are highlighted.

We constructed the maps of Sweden and its municipalities (Figs. 1A, 2 and 5A) from open-source shapefiles obtained from^25^ using the QGIS software^26^ version 3.2.

### Travel data

Travel patterns in the Swedish population have been recorded since the mid-1990s by interviewing representative selections of several thousand 6–84-year-old inhabitants. As part of this study, we obtained complete questionnaire data for the years 2011–2016^27^. The data contains posts on individuals’ daily commuting habits with partial and whole trips and the geographic origin and target at the DeSo level. Since DeSos are subdivisions of municipalities, we converted them to the municipality level.

Since this study considers the years from 2009 to 2015, we have used the 2011–2016 period to define the travel patterns for all parts of the analysis, including the years 2009 and 2010. We assume travel patterns did not change significantly between the years 2009 and 2011. Furthermore, while the sample size of the survey is adequate to calculate general statistics, it is too sparse to construct a travel network with temporal dynamics, even on a yearly level. Instead, we constructed an aggregated static network containing all feasible routes of infection. To partially compensate for the survey’s inadequacies, we made sure that the travel network represents all air and rail travel routes and connects all neighboring municipalities.

In order to include a variable representing travel frequency between the municipalities, we implemented the radiation model of Simini et al.^28^. This model uses the population size of areas and the distance between them to estimate the population movement between these areas.

### Epidemiological data

Our data set consists of all laboratory-verified cases of A(H1N1)pdm09 between May 2009 and December 2015, extracted from the SmiNet register of notifiable diseases, held by the Public Health Agency of Sweden. While the number of flu cases is regularly under-reported, the SmiNet database contains 16000 records, a reasonably large sample compared to other notifiable diseases. Due to confidentiality reasons, cases are anonymized, and addresses are aggregated at the DeSo level together with the date of diagnosis, age, and gender. To make sure the addresses represent the habitation at the time of diagnosis, Statistics Sweden (SCB) cross-referenced the register with a historical address register before anonymization. We obtained ethical approval for the data acquisition.

Consequently, the epidemiological data contains the number of daily reported cases at the DeSo level, which we converted to the municipality level. Since the available data covers an extended period from 2009 to 2015, we partitioned the case counts into six separate outbreaks corresponding to the 2009/2010, 2010/2011, 2011/2012, 2012/2013, 2013/2014, and 2014/2015 flu seasons. The size and timing of the outbreaks show considerable differences. The 09/10 swine flu pandemic covered a significant part of 2009 and lasted into the first weeks of 2010. In contrast, the 11/12 season saw almost no flu cases. Figure 1B shows the number of new cases in 2009 from week 37 to 50 in a few selected municipalities.

### Socioeconomic data

One of our main goals is to establish a relationship between socioeconomic factors and epidemic spreading. Since this study only considers Sweden, high-quality data is readily available from Statistics Sweden^29^, the organization responsible for coordinating the official statistics. Based on available statistics and previous studies^16–18,23^, we selected: 1. The median household income as an economic indicator. 2. The average number of children younger than 18 years per household to indicate family size. 3. The fraction of people receiving social aid to represent poverty in a municipality. 4. Population size and population density as the number of people per sq. km of land area. All the above statistics are available at the municipality level. Figure 2 shows the geographical distribution of indicators 1, 2, 3 and the population sizes of the municipalities of Sweden.

Both population density and population size have been used before to model epidemic spreading^16^. In a geographical setting, population density is preferred due to its independence from inequalities in the size of geographical areas. In contrast, using population size can be misleading if differences are more than minimal. However, the fine geographical resolution of the municipalities of Sweden and the lack of huge population centers (except Stockholm and Göteborg) allow us to use population size as a variable. We experimented with both density and size in our study. We found that density only has a small contribution to the timing of the epidemic events, while population size is a significant factor. Therefore, we chose to include population size.

### Meteorological data

Even though the exact mechanisms are unknown, the relationship between environmental temperature and humidity and seasonal influenza is well established in the literature^14–16,30^. As such, it is one of the key factors used in this study. Due to Sweden’s geographical position and the Gulf Stream effect, the country’s climate ranges from an oceanic climate in the far south to a subarctic climate in the far north, while central Sweden has a humid continental climate.

We obtained detailed meteorological data from the European Climate Assessment Dataset^31^. This database contains daily meteorological station observations covering Europe. Of the elements available in the database, we included mean temperature and relative humidity converted to absolute humidity as factors in this study. We show the averaged mean temperatures and absolute humidity of a few select municipalities on Fig. 1C,D.

## Method

The GIIM method uses a network to represent the geographical areas involved in the outbreak. The nodes of the network are the geographical areas themselves, while the links represent possible routes of infection. In the GIIM model, attributes (or weights) can be assigned to both the nodes and the edges of the network; these are important parts of the model and usually represent factors or determinants potentially related to disease spreading. The last and most important input of the model is a set of reference observations from an actual outbreak, which take the form of a time series of values on the same geographical resolution as the nodes of the network.

**Figure 3:**
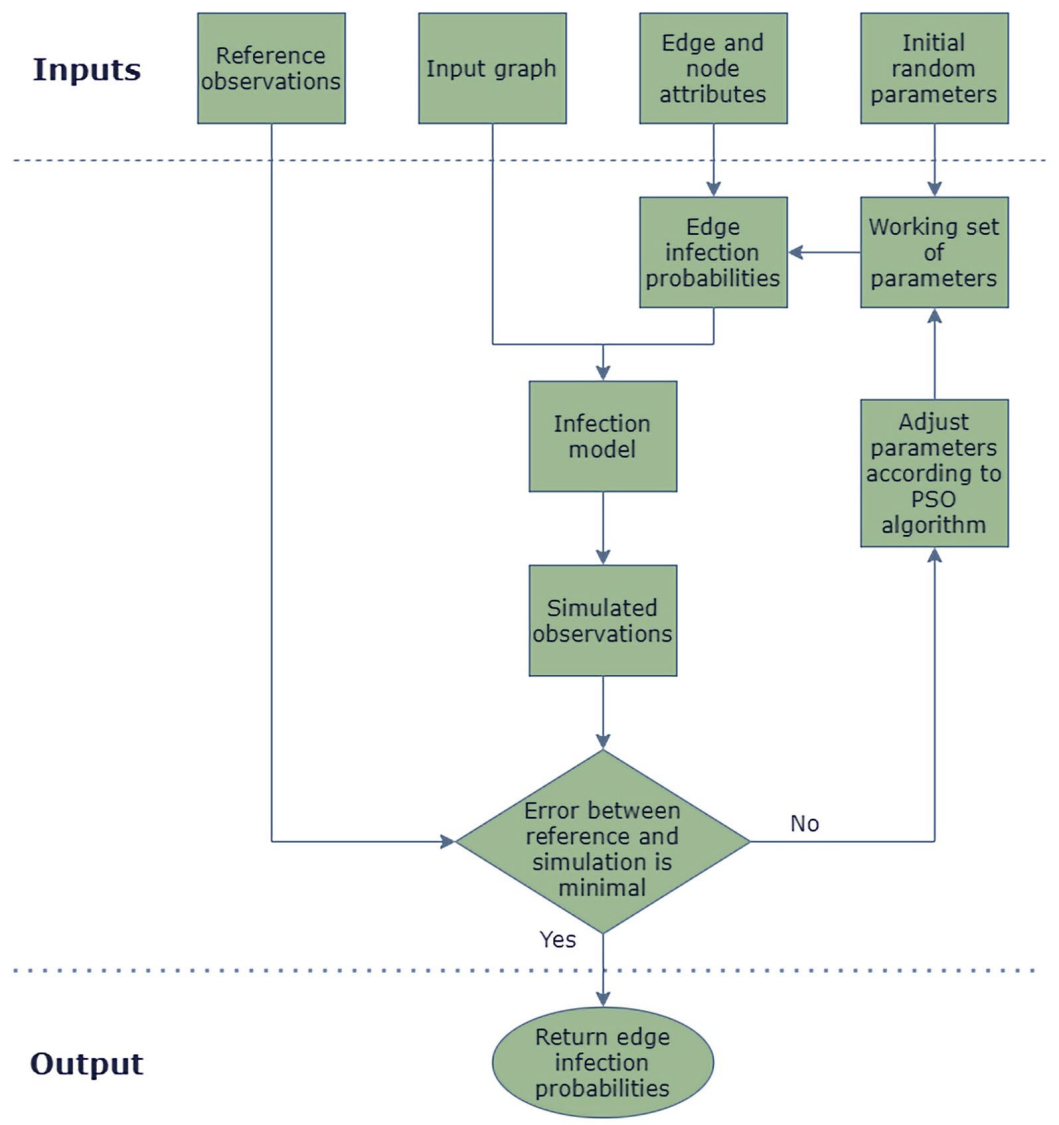
Flowchart of the GIIM model.

The goal of GIIM is to identify the transmission risks on the links of the network defined as a function of known attributes, and return the parameters of this function. The parameters are estimated with an iterative refinement approach. Starting from an initially random set of parameters, the method constructs a simulated outbreak and calculates a set of observations. These simulated observations are then compared to the reference observations and an error value is computed. Depending on the size of the error, the method either stops and returns set of edge infection probabilities, or it updates the set of parameters using the Particle Swarm Optimization algorithm. In latter case, the process is repeated until the error between the simulated and reference observations is minimal. Fig. 3 shows a flowchart of the method.

The GIIM method has been successfully applied to real-world problems^32^, including the modeling of the geographical spread and transmission of Zika in the Americas^23^. GIIM brings a novel approach to epidemic modeling because instead of merely simulating an outbreak, the method estimates an infection process’s parameters and properties using observations from an actual outbreak.

### Inputs

The GIIM method requires three inputs: an underlying network structure, attributes assigned to the nodes and edges of the network, and a set of observations on a real-life transmission process. The underlying network structure in this study represents the municipalities of Sweden. We denote graph *G* as *G*(*V*, *E*), where $V_G$ is a set containing all the vertices of the graph, while $E_G$ contains all the edges of the graph. We denote the edges of *G* as $e_{uv} \in E_G$ with *u* as the origin and *v* as the destination of the edge, where *u* and *v* are nodes of the network and edge $e_{uv}$ links node *u* to *v*. The nodes of the network represent the 290 municipalities of Sweden, and we define the edges of the network based on the travel survey introduced in the data section. We created a directed edge between two municipalities A and B if at least one individual traveled from A to B. To ensure that we represented all feasible travel paths in the network, we connected all neighboring municipalities. In this way, the edges indicate significant recorded travel between the pair of municipalities they connect. We denote this network $G_S$ and illustrate it in Fig. 1A.

#### Attributes

We represent the previously defined socioeconomic, travel, and meteorological factors as attributes assigned to the edges. All attributes are real values normalized between zero and one. The following attributes are assigned to each edge $e_{uv}$:

1. $F_{uv}$ the estimated amount of travel between municipalities *u* and *v* according to the radiation model.
2. $C^{t}_u$ the incidence of new flu cases reported at the origin municipality *u* in week *t*.
3. $T^{t}_v$ the mean temperature measured in the destination municipality *v* in week *t*.
4. $H^{t}_v$ the absolute humidity measured in the destination municipality *v* in week *t*.
5. $P_v$ the population size of the destination municipality *v*.
6. $I_v$ the median income per household in the destination municipality *v*.
7. $S_v$ the fraction of people receiving social aid in the destination municipality *v*.
8. $K_v$ the average number of children under 18 years of age per household in the destination municipality *v*.

Attributes marked with a time index *t* are dynamic. As such, their values change in time depending on the week of the outbreak. We build our model based on the 2009 flu pandemic. Therefore, all dynamic attributes refer to the 2009/2010 season unless noted otherwise. We only use data from the 2010–2015 period in the last part of our analysis, when we test our model’s predictive ability.

#### Attribute function

To reconstruct the observed outbreak using the network and its attributes, GIIM repeatedly runs a simulated infection process. The infection process requires transmission probabilities (also called edge infection probabilities) $w^{t}_{uv} \in [0,1], e_{uv} \in E_{G_S}$ assigned to the edges of the network. The flexibility of the GIIM model allows us to define these values as a function of known attributes. In this paper, we define the functions with the attributes listed above.

1 $$\begin{aligned} w^{t}_{uv} = A + \alpha F_{uv} + \beta C^{t}_u + \gamma T^{t}_v + \delta H^{t}_v + \zeta P_v + \eta I_v + \theta S_v + \kappa K_v \end{aligned}$$

The variables in Eq. (1) cover the attributes listed previously, while *A* denotes a constant. The optimization algorithm of GIIM estimates the coefficients of these functions. Note, that some of the attributes received a zero or close to zero weight during the optimization process. We left out these attributes from the final model. See the results section for a discussion of the final set of attributes and their coefficients.

#### Reference outbreak

The GIIM method seeks to estimate the parameters of an actual outbreak. Information about this outbreak comes in the form of reference observations. While observations can take different forms^20^, here we define the reference observations as a set of binary column vectors, where each row corresponds to a municipality, i.e. a node in the network, while each vector represents a given time period. The goal in this paper is to estimate the timing of the epidemic onset, therefore each value in these binary vectors indicates if the onset has already happened in the corresponding municipality in the corresponding time period. A value of 1 indicates that the epidemic onset already happened, while a 0 indicates that it has not happened yet. The week of the onset has a value of 1. We identified the week in which the epidemic onset happened in each municipality manually, by marking the week when the number of confirmed cases doubled compared to the previous week.

An example of a set of reference observations can be seen as follows.

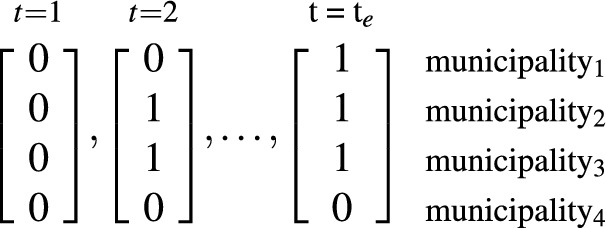



Each row corresponds to a municipality, *t* indicates a time period and there are $t_e$ time periods in total. The epidemic onset happened at $t = 2$ in municipality 2 and 3 and sometime later in municipality 1. Municipality 4 avoided the outbreak.

We model the spreading of influenza on a weekly basis, therefore each time period (and each binary vector) references a specific epidemic week. However, the timing of the epidemic seasons varies depending on the year. Table 1 lists the weeks in each season when there was a significant number of flu cases. We used these weeks to construct the reference observations. The 2009–2010 season had flu cases throughout the year, but we focus on the fall of 2009, where the largest portion of the outbreak took place.

**Table 1:** The start and end week of each epidemic season.

Season	Start week (year)	End week (year)
2009–2010	39 (2009)	50 (2009)
2010–2011	50 (2010)	7 (2011)
2011–2012	51 (2011)	9 (2012)
2012–2013	51 (2012)	7 (2013)
2013–2014	49 (2013)	10 (2014)
2014–2015	4 (2015)	13 (2015)

### Infection model

As part of its optimization process, GIIM relies on the repeated evaluation of a simulated infection process. GIIM’s flexibility allows it to be used with any infection model where the number of non-susceptible individuals is monotonically increasing^20^, but it was designed to work with models from the network-based SEIR model family. The SEIR compartmental model family was originally proposed by Kermack and McKendrick in 1927^33^, and has been widely used in the field of epidemic modeling ever since^22,34^. Members of the model family were adapted to networks first by Kleinberg et al.^37^, and later by multiple authors, e.g., to model global spreading of diseases^3^, to model the spreading of chikungunya on Reunion Island^35^, forecasting influenza in European countries^36^ or to model the establishment of Zika virus in the Americas during the pandemic^23^ in conjunction with GIIM.

In this study, we adopt the SI compartmental infection model defined for networks^20,37^, which was successfully used to model the first occurrence of Zika^23^ and the early stages of the COVID-19 pandemic^24^. Part of the more general SEIR infection model family, the SI model only has two states: susceptible (S) and infected (I), representing infectious nodes, which continuously try to infect their healthy neighbors, and susceptible nodes prone to infection. Each node of the network has a state during the process, which may change over time. We assign edge infection probabilities $w_{uv}^t \in [0,1]$ to all edges of the network. The *t* time index indicates that these probabilities may change their values depending on the discrete time scale of the process. However, $w_{uv}^t$ is strictly an input of the model and does not depend on the spreading process in any way. The infection process is iterative and takes place in a finite number of discrete time steps. In each iteration, a node may change its state depending on the state of its neighbors and the edge infection probabilities assigned to the edges connecting it to them. Nodes may change their states from susceptible to infected, but infected nodes stay infected until the end of the process. The total number of discrete time steps the process takes is limited to the number of weeks with reported new flu cases.

As in Ref.^20,23^, we define the SI infection model using a graph *G*(*V*, *E*) with edge infection probabilities $w^t_{uv}$ assigned to all of its edges and the initial set of infected nodes $A_0$. The rest of the nodes are in the susceptible state at the beginning of the process. Let $A_t \subseteq V_G$ be the set of infected nodes in iteration *t*. In each iteration *t* each infected node $u \in A_t$ tries to infect all its susceptible neighbors $v \in V_G \setminus A_t$ depending on the edge infection probability $w^t_{uv}$ of the edge connecting them. If the attempt is successful, *v* joins the set of infected nodes in the following iteration. If more than one node is trying to infect *v* in the same iteration, the attempts are made independently of each other in an arbitrary order within the same iteration. The process terminates naturally if all nodes reachable from the initially infected nodes with nonzero edge infection probabilities adopt the infected state, or when there are no more reported new flu cases.

The above process defines a single instance of an outbreak. Instead of binary values, GIIM requires for each node the likelihood of being in an infectious state for all time steps. To estimate the likelihood of infection for each node at each time step, we run the SI model *k* times and compute for each time step the fraction of instances where the nodes were infected. This approach is similar to the ones used in^38,39^. Following the observations in^39^ and our previous experiences in^23^, we set $k = 5000$. This setting provides good accuracy while reducing the overall runtime of the simulations. When we refer to the infection model’s output, we refer to the estimated likelihood values as opposed to the binary outputs of a single instance.

### The GIIM method

The GIIM method^20^ defines the problem of estimating edge infection probabilities as an optimization task. Its inputs include a network, several attributes on the nodes and the edges of the network, and a set of reference observations of an actual outbreak. GIIM provides an estimation of the observed outbreak by simulating one. Apart from the result of the simulation (which may be more detailed than the original), its output provides an assignment of edge infection probabilities and the relative importance of the attributes according to Eq. (1).

To define GIIM’s inputs, let $\vec {o}_t$ denote a vector containing observations on an infection process. Let $\vec {o}_t$ assign a value to all $v \in V_{G}$. Let $t \in T$ denote a discrete time stamp indicating the week in which the observation was taken, and *T* be the set of all time stamps. Let *O* denote the set of all observations $\vec {o}_t \in O$ for all $t \in T$. Let ${{\mathscr {I}}}$ denote the SI infection model introduced in the previous subsection, and $W_G: E_G \mapsto [0,1]$ be the initially unknown assignment of edge weights to the edges of the graph. Finally, let *Inf* be a procedure, which makes observations on infection process ${{\mathscr {I}}}$ at sample times *T*, taking place on graph *G* with assigned edge weights $w_{uv} \in W, e_{uv} \in E(G)$. We denote *Inf* as $O = Inf(G, W, {{\mathscr {I}}}, T)$.

#### Generalized inverse infection model

Given an weighted graph *G*, infection model ${{\mathscr {I}}}$, the set of sample times *T*, and reference observations $O = Inf(G, W, {{\mathscr {I}}}, T)$, we seek the edge infection probability assignment $W'$ such that the difference between *O* and $O' = Inf(G, W', {{\mathscr {I}}}, T)$ is minimal.

In this study, the set of reference observations *O* contains binary vectors indicating the timing of the epidemic onset, while the observations $O'$ generated by running the infection model ${{\mathscr {I}}}$ are real-valued. To compute the difference between *O* and $O'$, we employ ROC evaluation. We pairwise compare vectors $\vec {o}_t \in O$ and $\vec {o'}_t \in O'$ for all $t \in T$, calculating the AUC value for each pair and averaging over all pairs. The GIIM method uses an iterative refinement algorithm to solve the optimization task above. Starting from an initially random edge weight assignment, GIIM simulates an outbreak and compares the output with reference observations. Then it updates the edge weight assignments and repeats the process. The search algorithm uses the Fully Informed Particle Swarm Optimization method^21^, a multi-agent iterative optimization algorithm, which was previously shown to perform well with GIIM^20,23^. Algorithm 1 and Fig. 3 summarizes the GIIM algorithm.

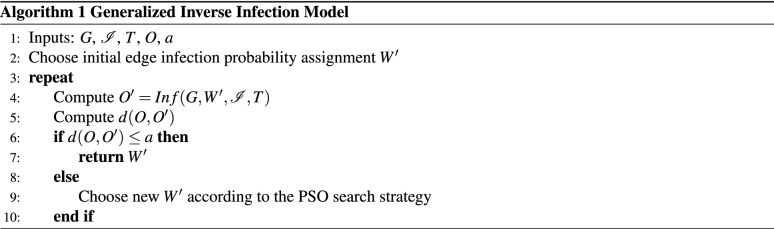



Estimating individual edges is difficult due to the size of most networks. To avoid this problem, GIIM defines the edge weights as a function of known attributes on the nodes or edges. This way, the goal of the optimization task is to find the coefficients of this function. GIIM also makes it possible to define dynamic attributes or edge weights, indicating that their value changes in time. In practice, this means that the edge weight or attribute is a function of *t*, a discrete time stamp corresponding to the actual iteration of the infection model.

The general form of the edge function can be written as $w_{uv}^t = g(f_1(a_1^t(e_{uv}), \vec {c_1}),$ $f_2(a_2^t(e_{uv}), \vec {c_2}), \dots , f_{\ell }(a_{\ell }^t(e_{uv}), \vec {c_{\ell }}), \vec {c_g})$ for all $e_{uv} \in E_{G_A}$, where $a_i^t(e_{uv})$ represents the *i*-th attribute on edge $e_{uv} \in E_{G}$ at iteration *t* of the infection process, $\ell$ denotes the number of available attributes, $f_1, \dots , f_{\ell }$ and *g* are functions and $\vec {c_1}, \dots , \vec {c_{\ell }, \vec {c_g}}$ are coefficients of functions $f_1, \dots , f_{\ell }, g$. This formulation is easy to implement and allows us to assign different functions to different attributes, while the role of function *g* is to aggregate and normalize the results of the individual attribute functions to ensure they fall between 0 and 1. The value $w_{uv}^t$ denotes the edge weights and *C* the set of all coefficient vectors. Using the function-based alternative greatly simplifies the optimization task, reducing the number of values we have to estimate from $|W|=|E_G|$ to |*C*|. However, its main advantage is that instead of providing a single individual value for each edge, it allows us to explore the relationship between the factors potentially related to the outbreak and the outbreak itself.

Equation (1) defines the edge functions used in this study. We trim the weighted sums above 1 and below 0 by taking $w_{uv}^t = MAX(0, MIN(1, \sum _{i = 1}^{\ell } f(a_i^t(e_{uv}), c_i) + c_g)))$. To reduce the solution space of the PSO method and to make the results of individual test runs comparable, we bound all parameters between $-0.5$ and 0.5, except *A*, which we bound between $-1$ and 1.

### Ethics approval and consent to participate

All data were anonymized to be not retraceable to individuals by the Public Health Agency of Sweden before being accessed by the research team. Approval was granted by the Regional Ethical Committee in Umeå on 2015-10-20.

## Results and discussion

We start our analysis by studying the spreading of H1N1 in the autumn of 2009 between weeks 37 and 50, corresponding to a period from mid-September to mid-December. We model the outbreak on a weekly basis and set up the input files of GIIM as described in the Inputs section. To partially account for the summer wave of infections in 2009, we select the municipalities of Stockholm, Malmo and Gothenburg as infected in the starting week of the analysis because these municipalities contained the most infected individuals during the summer wave. We use the attribute function defined in Eq. (1) with the socioeconomic, travel and meteorological factors introduced in the Inputs section. To compensate for the model’s stochastic nature, we ran the algorithm 20 times with the same set of inputs and computed the mean and variance of the results.

### Risk factors

**Figure 4:**
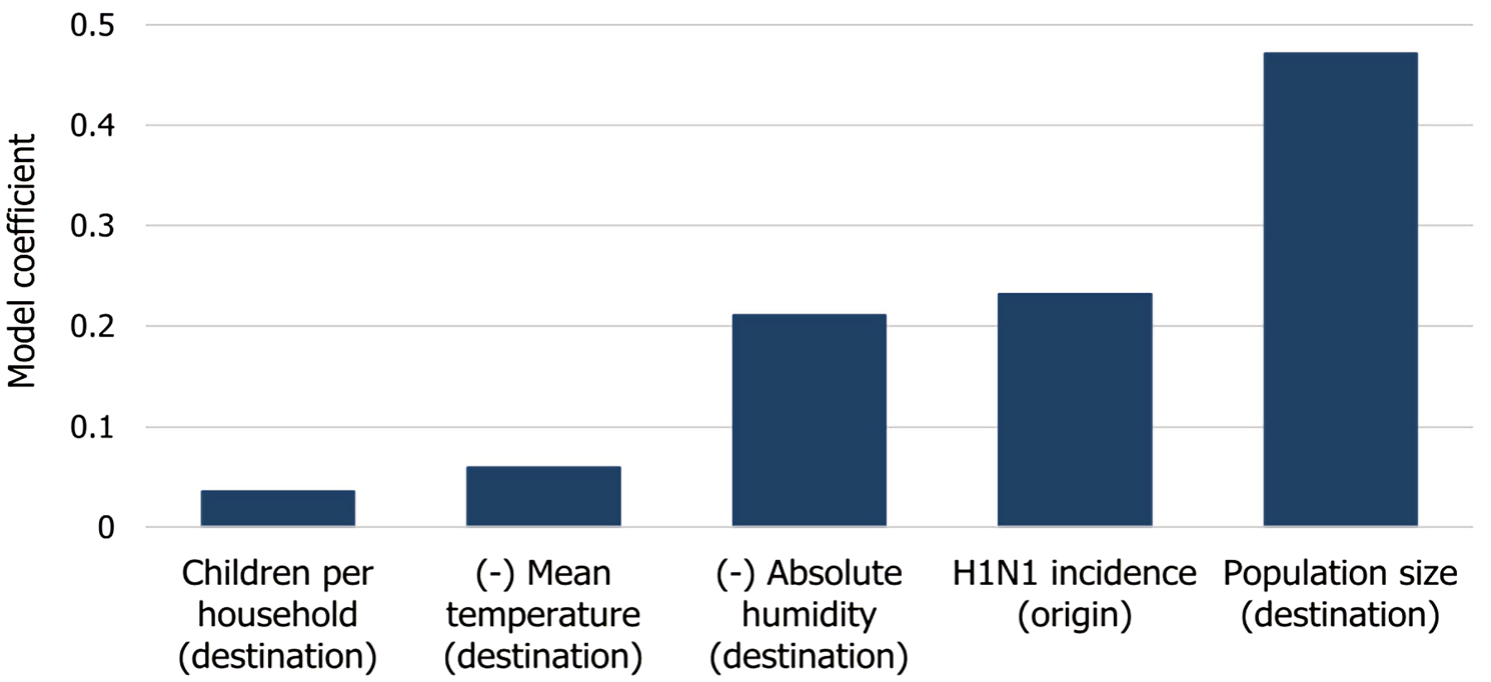
Coefficients assigned to the risk factors by GIIM.

To evaluate the relative contributions of the travel, socioeconomic, and meteorological factors on the timing of the epidemic onset in the municipalities of Sweden, we experimented with multiple combinations and functions. While we started our analysis with the function shown in Eq. (1), several of our input attributes received a coefficient close to zero. These were travel frequency ($F_{uv}$), household income ($I_v$) and social aid ($S_v$). We excluded these variables from the final model in Eq. (2). Figure 4 shows the mean of the coefficients assigned to the remaining risk factors, while Table 2 shows the mean, the minimum, the maximum and the standard deviation of these factors.

2 $$\begin{aligned} w^{t}_{uv} = 0.001 + 0.2317 C^{t}_u - 0.0594 T^{t}_v - 0.2106 H^{t}_v + 0.4712 P_v + 0.0357 K_v \end{aligned}$$

**Table 2:** The mean, the minimum, the maximum and the standard deviation of coefficient assigned to the risk factors by GIIM.

Risk factor	Mean	Minimum	Maximum	SD
Population size (destination)	0.4712	0.4293	0.4981	0.0207
H1N1 incidence (origin)	0.2317	0.2033	0.2667	0.0208
Absolute humidity (destination)	− 0.2106	− 0.3122	− 0.0416	0.0917
Mean temperature (destination)	− 0.0594	− 0.0636	− 0.0543	0.0034
Children per household (destination)	0.0357	0.0321	0.0401	0.0025

The weights in Eq. (2) represent each risk factor’s relative contribution to the transmission risk between the municipalities. The standard deviation of the coefficients across test runs is small except for absolute humidity. More importantly, the relative importance of the risk factors remains robust.

The most significant factor in the timing of the epidemic onset is the population size of the municipalities, indicating that the disease reaches large population centers first and then spreads to the countryside. This result is in line with observations in the US and UK, where both population size and the more commonly used population density were already established as important contributors to this phenomenon^8^. Our results indicate that the importance of large population centers is far greater than identified in^13^, possibly because of the higher spatial resolution of our model in Sweden.

Another significant risk factor is the incidence of H1N1 cases in the municipalities on the origin of the links. This coefficient is closely tied to the network-based structure of our model, which is similar to agent-based approaches^6,7^ and underlines the spatial structure of the spreading process, which we discuss in the next subsection.

The contribution of the amount of travel between municipalities was close to zero in our initial model, thus the parameter was omitted from Eq. (2). This result is in line with the observations in^13^. The exact role of this indicator is debated in the literature. Some studies identify it as a minor factor^5,16,23^, while others emphasize its effect in local or global transmission^7,8^. One possible explanation for its lack of importance here, is that our study only considers a relatively small geographical area, a single country having a well developed transportation infrastructure. Our results are also in line with the conclusions in^12,23^, which states that the presence of a few infected travelers is enough to spread the disease to different regions.

Meteorological factors such as temperature and humidity are known to play a critical role in the appearance of influenza^12,16^. According to our model, a decrease in absolute humidity is one of the main driving factors in H1N1 spreading, while a drop in temperature provides a smaller contribution. This result confirms the findings of Skog et al.^12^, but contradicts the findings of Morris et al.^13^, which identified no such relationship. We provide two explanations for this: First, our model offers a much higher spatial resolution for Sweden than the model in^13^. Second, the high-resolution results of Morris et al. apply to Norway, which has a different climate closer to the Norwegian Sea and the North Atlantic Current with the Scandinavian Mountains separating the two countries.

Perhaps due to the relative homogeneity of a single country, we found that the effect of the socioeconomic variables on the transmission risk between municipalities to be minimal. The average number of school-age children per household, which is a well-known risk factor^17^, showed the most significant influence. The influence of both income and social aid is close to zero in our model. However, median income and social aid at the municipality level might not be sufficiently sensitive indicators to measure the inequalities in Swedish society.

We conclude that the three variables that influence the timing of the epidemic onset the most are population size, the number of cases in neighboring municipalities and a decrease in the absolute humidity of the environment. This result is in line with the spreading mechanism of agent-based simulations where the outbreak travels from region to region after infecting a certain amount of people locally, favoring population centers^6,7^. Temperature also has a small negative effect, while school-aged children have a small positive effect.

### Exportation, importation and route level risk

**Figure 5:**
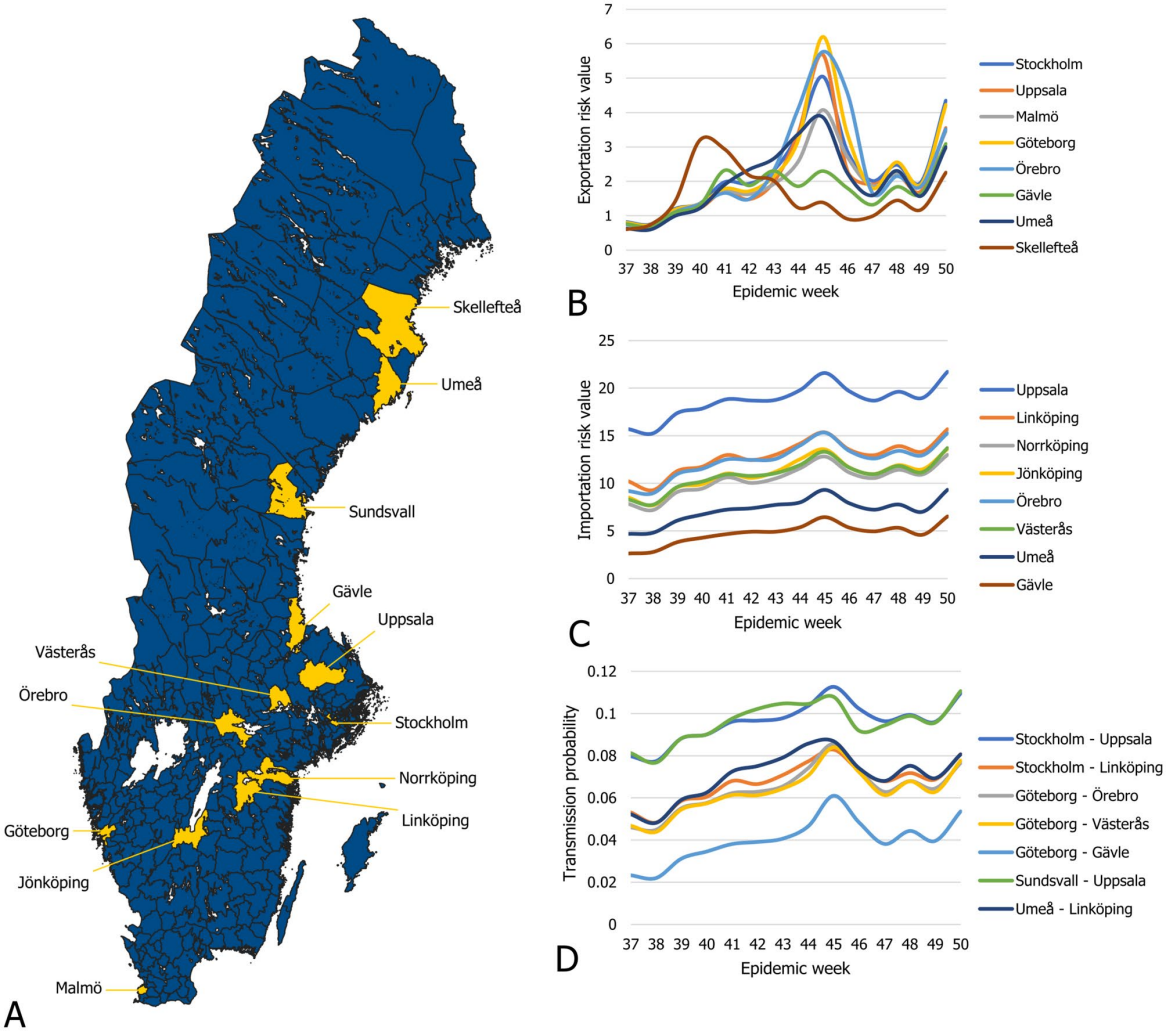
(**A**) Municipalities on (**B**–**D**) are highlighted on the map. (**B**) Exportation risk values of selected municipalities. (**C**) Importation risk values of selected municipalities. (**D**) Transmission probabilities of selected links.

The edge infection or transmission probabilities between the municipalities of Sweden are among the outputs of GIIM. It is possible to define a node-based import and export risk weight by aggregating the edge-based risk values on the in- and out-edges of each node. This value is also known as node strength. These values are not probabilities but risk indicators of importing or exporting the disease from or to a municipality. They represent a relative weight that allows us to rank the municipalities. Figure 5 shows exportation, importation and route level risk for a few select municipalities, while Fig. 5A shows their location in Sweden.

Figure 5B illustrates the time-dependent exportation risk values for some of the highest risk municipalities in onset estimation. Large population centers appear at the top of the list. While Göteborg and Stockholm remain at the top, Malmö only stays in the top 20 exporters during the initial weeks, with its relevance decreasing even more later in the outbreak. Other larger cities include Linköping, Jönköping and Uppsala, although their relevance fades in time except for Uppsala. Several of the larger cities of northern Sweden, such as Skellefteå, Umeå, and Gävle, are top exporters until mid-November, partially confirming the observations in^12^.

Figure 5C illustrates the time-dependent importation risk values for some high-risk municipalities in onset estimation. This ranking is more static than the export ranking, with large population centers remaining high risk until the end of the outbreak. These cities include Linköping, Jönköping, Norrköping, Uppsala, Örebro and Västerås, together with a few northern municipalities such as Umeå, Gävle and Sundsvall. In contrast to the export risk ranking, Stockholm county and Västra Götaland are not top-import municipalities. However, our model only considers domestic travel patterns in Sweden, therefore the risk of importation from international travellers is not included in our analysis. This fact may explain the seemingly low risk of importation in Stockholm.

Due to a large number of edges in the graph, it is difficult to highlight the individual links most important to the outbreak. Figure 5D shows some of these links. For example, early in the outbreak, links connecting to Uppsala appear most prominently among links to Umeå, Linköping, Örebro and Västerås. This pattern remains until the end of the outbreak. Starting from mid-October, links connecting the municipalities of Northern Sweden appear at the top of the edge-based risk ranking and stay there for several weeks until mid-November. At the end of our observation period, links connecting larger population centers in South and Central Sweden dominate the top of the ranking.

Our observations on the timing of the epidemic onset of H1N1 in the fall of 2009 follow a loose geographical pattern. Following the smaller summer wave of infections, the disease stays in the largest cities. From September, it begins slowly spreading to the other larger population centers of South and Central Sweden and a few cities in Northern Sweden. A massive burst of the outbreak happens in mid-October, reaching the countryside in South and Central Sweden and moving north along the coast of the Gulf of Bothnia, infecting the larger population centers on its way. After peaking in most of the country in early and mid-November, the outbreak first ends in the north and finally in the largest cities where it began.

### Model accuracy

**Figure 6:**
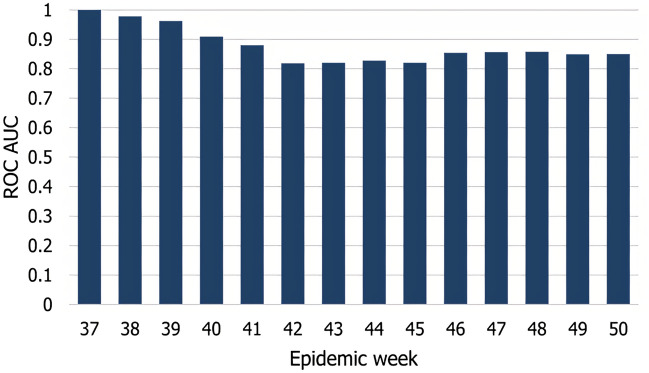
Model accuracy (ROC AUC) for all weeks of the observation period.

We evaluated the accuracy of our model by independently computing the ROC AUC value for each week of the estimation process. The averaged AUC value for all 14 weeks of the observation period was 0.875, indicating a good fit. Figure 6 shows individual AUC values for all weeks. The estimation is easy during the first weeks due to the small number of positive examples. In most municipalities, the fall H1N1 outbreak started and peaked in weeks 42–45. In these weeks, the accuracy of the estimation process drops to around 0.82, indicating some uncertainty in the exact timing of the onset, stabilizing around 0.85 in the second half of the observation period.

### Model predictability

To demonstrate the robustness of our approach, we use the attribute function with estimated coefficients from the 2009 outbreak in conjunction with the infection model to predict the timing of the epidemic onset in the influenza outbreaks in epidemic seasons 10/11, 12/13, 13/14, 14/15. We omitted season 11/12 from our analysis due to the small number of H1N1 cases in that season. We updated our dynamic input attributes to match the actual temperature and humidity values in the periods. We also defined the infection sources for each of the target seasons and estimation types by selecting the three municipalities with the earliest appearance of the disease.

To test the accuracy of our predictions, we set up the reference observations for our four target seasons in the same way as for the 09/10 season (for observation periods of these seasons, see Table 1). After running the infection model for each season, we compared their output with the corresponding reference observations by computing the ROC AUC metric.

**Figure 7:**
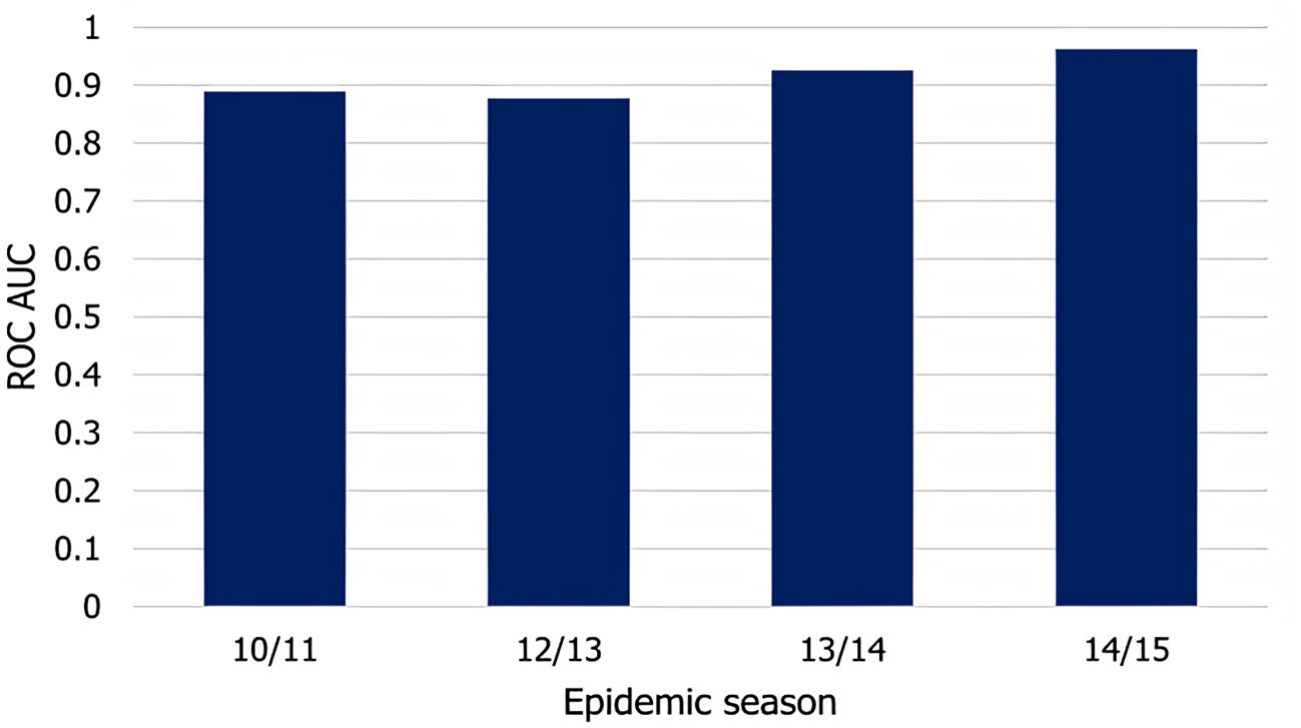
Predictive accuracy for epidemic seasons 10/11, 12/13, 13/14, 14/15.

As shown in Fig. 7, the average AUC values for all successive seasons stay close or even exceed the accuracy we have seen in the 2009 pandemic. The individual AUC values present a similar picture, although the trends inside epidemic seasons differ somewhat from those in Fig. 6, with accuracy slowly decreasing as the outbreak progresses but stabilizing well above 0.8. The predictive accuracy is higher in the 13/14 and 14/15 seasons than in the first two. While the weakening immunity granted to the population by the 09/10 pandemic may explain this trend, our results do not provide conclusive evidence to confirm this hypothesis.

## Conclusions

According to our findings, the spreading of H1N1 in Sweden was mainly driven by large population centers, the presence and size of outbreaks in neighboring municipalities and the meteorological factors humidity and temperature. Confirming observations in^13,23^, the amount of travelers is not an important factor. This result may be due to the relatively small geographical scope of the study and the well-developed infrastructure of the country. Supporting existing results on the effect of meteorological factors in influenza spreading^12,16^, absolute humidity plays a critical role in our model, while mean temperature contributes less. The only other socioeconomic indicator that contributes noticeably to our model is the number of children per household, confirming the observations in^17^. The effect of the rest of the socioeconomic factors, income and social aid, is close to zero. However, the spatial resolution of our model might be too low to identify their contribution.

Compared to other studies discussing the role of socioeconomic, climate and travel indicators in influenza spreading in the Nordic countries, we confirm most of the observations of Skog et al.^12^ and some of Morris et al.^13^. Due to the radically different methodology used in these studies, a more direct comparison is not within the scope of this paper. We also extended these previous works by examining the effect of a larger set of indicators and providing an analysis of the most critical travel routes involved in the spreading of influenza.

While our model is constructed based on the 2009 pandemic, we can make accurate predictions on the timing of the selected epidemic events in the following seasons. Therefore, our model can be used as a real-time decision support tool advising on resource allocation and surveillance. Furthermore, while our study only considers H1N1 spreading, it can be adapted to model other influenza strains or respiratory infections with a similar transmission mechanism.

## Data Availability

The input and output data used in this paper can be found at http://snd.gu.se/en/catalogue/study/2021-282

## Code availability

The implementation of the proposed algorithm is available on request from the authors.
